# Neurodevelopmental Disorders across the Lifespan: A Neuroconstructivist Approach. Edited by Emily K. Farran and Annette Karmiloff-Smith, Oxford University Press, 2012; 394 pages. Price: £49.99, ISBN 978-0-19-959481-8

**DOI:** 10.3390/brainsci3010084

**Published:** 2013-01-15

**Authors:** Shu-Kun Lin

**Affiliations:** MDPI AG, Kandererstrasse 25, CH-4057 Basel, Switzerland; E-Mail: lin@mdpi.com

The following paragraphs are reproduced from the website of the publisher [1].

- The first book to consider atypical development across multiple levels (genes, brain, behavior, environment), encouraging readers to think dynamically and developmentally, rather than examining static snapshots of neurodevelopmental disorders.
- Provides the most comprehensive review of development across cognitive domains (and their interactions), making clinicians more sensitive to looking for underlying cognitive and neural differences even when behavioral scores are in the normal range.
- Considers development from infancy to adulthood, encouraging the reader to think about the importance of development in understanding neurodevelopmental disorders, for example, by considering the impact that differences in low-level processes in infancy can have on later developing cognitive processes.

Nowadays, it is widely accepted that there is no single influence (be it nature or nurture) on cognitive development. Cognitive abilities emerge as a result of interactions between gene expression, cortical and subcortical brain networks, and environmental influences. In recent years, our study of neurodevelopmental disorders has provided much valuable information on how genes, brain development, behavior, and environment interact to influence development from infancy to adulthood.

This is the first book to present evidence on development across the lifespan across these multiple levels of description (genetic, brain, cognitive, environmental). In the book, the authors have chosen a well-defined disorder, Williams syndrome (WS), to explore the impact of genes, brain development, behavior, as well as the individual's environment on development. WS is used as a model disorder to demonstrate the authors’ approach to understanding development, whilst being presented in comparison to other neurodevelopmental disorders—Autism, Developmental Dyscalculia, Down syndrome, Dyslexia, Fragile X syndrome, Prader-Willi syndrome, Specific Language Impairment, Turner syndrome—to illustrate differences in development across neurodevelopmental disorders.

Williams syndrome is particularly informative for exploring development: Firstly, it has been extensively researched at multiple levels: genes, brain, cognition and behavior, as well as in terms of the difficulties of daily living and social interaction. Secondly, it has been studied across the lifespan, with many studies on infants and toddlers with WS as well as a large number on children, adolescents and adults. The authors also explore a number of domain-general and domain-specific processes in the verbal, non-verbal and social domains, across numerous neurodevelopmental disorders. This illustrates, among other factors, the importance of developmental timing, *i.e.*, that the development of a cognitive skill at a specific timepoint can impact on subsequent development within that domain, but also across domains. In addition, the authors discuss the value of investigating basic-level abilities from as close to the infant start-state as possible, presenting evidence of where cross-syndrome comparisons have shed light on the cascading impacts of subtle similarities and discrepancies in early delay or deviance, on subsequent development.

Designed such that readers with an interest in any neurodevelopmental disorder can gain insight into the intricate dynamics of cognitive development, the book covers both theoretical issues and those of clinical relevance. It will be an invaluable reference for any researcher, clinician or student as well as interested parents or teachers wishing to learn about neurodevelopmental disorders from a developmental framework.

Readership: Researchers studying development and developmental disorders—including psychologists, neuroscientists, geneticists.

**Table of Contents**
List of Contributors
Introduction
Williams Syndrome: A Model for the Neuroconstructivist Approach
**Section 1 Cognition, Brain, Genes**
  1. Michael S. C. Thomas, Harry R. Purser, Jo van Herwegen: Cognition: The Developmental Trajectories Approach
  2. Annette Karmiloff-Smith: Brain: The Neuroconstructivist Approach
  3. Lucy R. Osbourne: Genes: The Gene Expression Approach
**Section 2 Clinical and Practical Outcomes**
  4. Kay Metcalfe: Clinical Profile: Diagnosis and Prognosis
  5. Chris Stinton & Patricia Howlin: Adult Outcomes and Integration into Society
**Section 3 Domain-General Processes**
  6. Kate Breckenridge, Jan Atkinson, Oliver Braddick: Attention
  7. Dagmara Annaz, Anna Ashworth: Sleep-Related Learning
  8. Stefano Vicari, Deny Menghini: Memory
  9. Kerry D. Hudson, Emily K. Farran: Executive Function and Motor Planning
**Section 4 Domain-Specific Processes**
  10. Carolyn B. Mervis, Angela E. John: Language Precursors and Early Language
  11. Vesna Stojanovik: Later Language
  12. Emily K. Farran, Susan C. Formby: Visual Perception and Visuo-Spatial Cognition
  13. Jan Atkinson, Oliver Braddick: Spatial Cognition, Visuo-Motor Action and Attention
  14. Debbie M. Riby: Face Processing and Social Interaction
  15. Ruth Campos, Maria Sotillo: Mental State Understanding and Social Interaction
  16. Joanne S. Camp, Emily K. Farran, Annette Karmiloff-Smith: Numeracy
  17. Yvonne M. Griffiths: Literacy
**Section 5 The Neuroconstructivist Approach to Domain-General and Domain-Specific Processes**
  18. Ann Steele, Janice Brown, Gaia Scerif: Integrating Domain-General and Domain-Specific Developmental Processes: Cross-Syndrome, Cross-Domain Dynamics
**Conclusion**
  Annette Karmiloff-Smith & Emily K. Farran: Future Theoretical and Empirical Directions within a Neuroconstructivist Framework
Author Index
Subject Index

* *Editor’s Note*: The brief summary and the contents of the books are reported as provided by the authors or the publishers. Authors and publishers are encouraged to send review copies of their recent books of potential interest to readers of *Brain Sciences* to the Publisher (Dr. Shu-Kun Lin, Multidisciplinary Digital Publishing Institute (MDPI), Kandererstrasse 25, CH-4057 Basel, Switzerland. Tel.: +41-61-683-77-34; Fax: +41-61-302-89-18; E-Mail: lin@mdpi.com). Some books will be offered to the scholarly community for the purpose of preparing full-length reviews.
